# Intraamniotic Inflammation in Women with Preterm Prelabor Rupture of Membranes

**DOI:** 10.1371/journal.pone.0133929

**Published:** 2015-07-24

**Authors:** Ivana Musilova, Radka Kutová, Lenka Pliskova, Martin Stepan, Ramkumar Menon, Bo Jacobsson, Marian Kacerovsky

**Affiliations:** 1 Department of Obstetrics and Gynecology, Charles University in Prague, Faculty of Medicine Hradec Kralove, University Hospital Hradec Kralove, Hradec Kralove, Czech Republic; 2 Institute of Clinical Biochemistry, University Hospital Hradec Kralove, Hradec Kralove, Czech Republic; 3 Department of Obstetrics and Gynecology, Sahlgrenska Academy, Gothenburg, Sweden; 4 Department of Genes and Environment, Division of Epidemiology, Norwegian Institute of Public Health, Oslo, Norway; 5 Department of Obstetrics and Gynecology, Division of Maternal-Fetal Medicine & Perinatal Research, The University of Texas Medical Branch at Galveston, Galveston, Texas, United States of America; 6 Biomedical Research Center, University Hospital Hradec Kralove, Hradec Kralove, Czech Republic; Xavier Bichat Medical School, INSERM-CNRS - Université Paris Diderot, FRANCE

## Abstract

**Objective:**

To characterize subgroups of preterm prelabor rupture of membranes (PPROM) and short-term neonatal outcomes based on the presence and absence of intraamniotic inflammation (IAI) and/or microbial invasion of the amniotic cavity (MIAC).

**Methods:**

One hundred and sixty-six Caucasian women with singleton pregnancies were included in this study. Amniotic fluid samples were obtained by transabdominal amniocentesis (n=166) and were assayed for interleukin-6 levels by a lateral flow immunoassay. The presence of *Ureaplasma* species, *Mycoplasma hominis*, *Chlamydia trachomatis*, and 16S rRNA was evaluated in the amniotic fluid. IAI was defined as amniotic fluid IL-6 values, measured by a point of care test, higher than 745 pg/mL.

**Results:**

Microbial-associated IAI (IAI with MIAC) and sterile intraamniotic inflammation (IAI alone) were found in 21% and 4%, respectively, of women with PPROM. Women with microbial-associated IAI had higher microbial loads of *Ureaplasma* species in the amniotic fluid than women with MIAC alone. No differences in the short-term neonatal morbidity with respect to the presence of microbial-associated IAI, sterile IAI and MIAC alone were found after adjusting for the gestational age at delivery in women with PPROM.

**Conclusions:**

Microbial-associated but not sterile intraamniotic inflammation is common in Caucasian women with PPROM. The gestational age at delivery but not the presence of inflammation affects the short-term neonatal morbidity of newborns from PPROM pregnancies.

## Introduction

Preterm prelabor rupture of membranes (PPROM) is defined as a rupture of fetal membranes with leakage of amniotic fluid prior to 37 weeks of gestation. PPROM represents a serious pregnancy complication that is responsible for approximately one-third of all preterm deliveries. Recent studies suggest its mainly non-infectious etiology with inflammation as the major underlying pathophysiology [1–5]. However, it is unclear whether inflammation is a cause or consequence of PPROM and how it is linked to PPROM risk factors [6,7].

PPROM is often complicated by microbial invasion of the amniotic cavity (MIAC) and intraamniotic inflammation (IAI), resulting in the development of histological chorioamnionitis. MIAC, which is predominated by *Ureaplasma* species, complicates approximately 25–40% of PPROM, depending on the gestational age at sampling, ethnicity, and detection technique [8,9]. The presence of bacteria in the amniotic fluid is postulated to activate an intraamniotic innate immune response through the system of pattern recognition receptors, resulting in microbial-associated IAI [8–11]. Moreover, the intensity of IAI depends on the type of bacteria and the microbial load [12–14]. Therefore, the presence of a small amount of bacteria with a low virulent potential, such *Ureaplasma* species, in the amniotic is unlikely to elicit IAI and be associated with worse pregnancy and neonatal outcomes. This scenario is considered colonization of the amniotic fluid [15]. On the other hand, some endogenous mediators called alarmins (e.g., high mobility group box-1 protein) are released into the amniotic fluid and can trigger intraamniotic inflammation through the same system of pattern recognition receptors as in the infectious scenario [5,16]. This leads to the development of sterile IAI (the presence of IAI without any proven microorganism in the amniotic fluid).

However, the diagnosis of microbial-associated or sterile IAI is very often time consuming and costly due to the need for non-cultivation techniques to prove or rule out the presence of MIAC. Currently, the use of non-cultivation techniques to diagnose MIAC should be considered a gold standard because many amniotic fluid microorganisms are difficult to cultivate [10,17].

A pioneering study by Romero et al. has shown that the incidences of microbial-associated and sterile IAI in pregnancies complicated by PPROM are equal (approximately 30%) [3]. Because racial disparity can affect the levels of intraamniotic mediators, resulting in a different rate of sterile IAI, it would be of interest to evaluate whether this distribution of IAI subtypes is observed in a European population consisting of a majority of Caucasian women [18,19]. Moreover, there is a lack of evidence for whether the presence of both subtypes of IAI and MIAC alone affect short-term neonatal morbidity.

Therefore, the main aim of this study was to characterize the subgroups of women with PPROM with respect to the presence of IAI and/or MIAC. The second aim was to evaluate the selected aspects of short-term neonatal morbidity with respect to the presence of IAI and/or MIAC. A positive amniotic interleukin (IL)-6 by point-of-care testing was used as the definition of IAI due to its proven prognostic and clinical utility [20–25].

## Materials and Methods

The Institutional Review Board at University Hospital Hradec Kralove (March 19, 2008; no. 200804 SO1P) approved the study and written informed consent was obtained from all the participants. Between January 2012 and July 2014, a prospective cohort study was conducted on pregnant women at gestational ages 24+0 or 36+6 weeks, who were admitted to the Department of Obstetrics and Gynecology, University Hospital Hradec Kralove, Czech Republic. Pregnant women with singleton pregnancies complicated by PPROM and with a maternal age ≥ 18 years were invited to participate in the study. Women with signs of fetal growth restriction, the presence of either congenital or chromosomal fetal abnormalities, gestational or pre-gestational diabetes, gestational hypertension, preeclampsia, signs of fetal hypoxia, or significant vaginal bleeding were excluded from the study.

Gestational ages were established by first-trimester fetal biometry. In the Czech Republic, women with PPROM at less than 34 weeks of gestation are treated with corticosteroids to accelerate lung maturation (two doses of 14 mg of betamethasone, administered intramuscularly 24 hours apart), tocolytics for 48 hours, and antibiotics (prophylactic parenteral azithromycin were given at admission for maximum 7 days), whereas no treatment except antibiotics is initiated to delay delivery after 34 weeks. The management of PPROM in the Czech Republic is active (except at < 28 gestational weeks); the induction of labor or an elective cesarean section is initiated no later than 72 hours after the rupture of the membranes, depending on the gestational age of the pregnancy, fetal status, and maternal serum levels of C-reactive protein.

PPROM was diagnosed by examination with a sterile speculum to verify the pooling of amniotic fluid in the vagina, and when necessary, it was confirmed by the presence of insulin-like growth factor binding proteins (ACTIM PROM test; MedixBiochemica, Kauniainen, Finland) in the vaginal fluid.

Ultrasound-guided transabdominal amniocentesis was performed at admission, but before the administration of antibiotics, corticosteroids, and tocolytics, approximately 2–3 mL of amniotic fluid was aspirated. A total of 100 μL of non-centrifuged amniotic fluid was used for the bedside assessment of IL-6 levels. The remaining amniotic fluid was immediately transported to the microbiology laboratory for polymerase chain reaction (PCR) testing for *Ureaplasma* species, *Mycoplasma hominis*, and *Chlamydia trachomatis* as well as for evaluating 16S rRNA.

This study was approved by the Institutional Review Board committee (March 19, 2008; No 200804 SO1P), and informed consent was received from all participants.

### Amniotic fluid IL-6 levels

The IL-6 levels were assessed with a lateral flow immunoassay Milenia QuickLine IL-6 using the Milenia POCScan Reader (Millenia Biotec, GmbH, Giessen, Germany). The measurement range was 50–10000 pg/mL. The intraassay and interassay variations were 12.1% and 15.5%, respectively.

### Definition of IAI

Based on recent studies, IAI in PPROM pregnancies was defined as amniotic fluid IL-6 values, measured by a point of care test, higher than 745 pg/mL [24–26].

### Detection of *Ureaplasma* species, *Mycoplasma hominis*, and *Chlamydia trachomatis*

DNA was isolated from the amniotic fluid with a QIAamp DNA Mini Kit (QIAGEN, Hilden, Germany) according to the manufacturer’s instructions (using the protocol for the isolation of bacterial DNA from biological fluids). Real-time PCR was performed on a Rotor-Gene 6000 instrument (QIAGEN, Hilden, Germany) with the commercial kit AmpliSens C. trachomatis/Ureaplasma/M. hominis-FRT (Federal State Institution of Science, Central Research Institute of Epidemiology, Moscow, Russia) to detect the DNA from *Ureaplasma* species, *Mycoplasma hominis*, and *Chlamydia trachomatis* in a common PCR tube. As a control, we included a PCR run for beta-actin, a housekeeping gene, to examine the presence of inhibitors of the polymerase chain reaction. The amount of *Ureaplasma* species DNA in copies/mL was determined by an absolute quantification technique employing an external calibration curve. Plasmid DNA (pCR4, Invitrogen) was used for the preparation of the calibration curve [12,13].

### Detection of other bacteria in the amniotic fluid

Bacterial DNA was identified by PCR targeting the 16S rRNA gene with the following primer pars: 5`-CCTACGGGNGGCWGCAG-3`(V3 region), 5`-GACTA CHVGGGTATCTAATCC-3`(V4 region), and 5`-AGGAGGTGATCCAACCGCA-3`(V7 region), 5`-GGTTAAGTCCCGCAACGAGCGC-3`(V9 region) [27,28]. Each individual reaction contained 5 μL of target DNA, 500 nM of forward and reverse primers and Mastermix 16S Basic (Molzyme, Bremen, Germany) in a total volume of 25 μL. The amplification was performed in a 2720 Thermal Cycler (Applied Biosystems, Foster City, CA, USA). The products were visualized on an agarose gel together with the beta-actin control (309 bp). Positive reactions yielded products of 452 bp, which were subsequently analyzed by sequencing. The 16S PCR products were cleaned and used in sequencing PCR reactions utilizing the above primers and the BigDye Terminator kit, version 3.1. Bacteria were then typed using the sequences obtained in BLAST and SepsiTest BLAST.

### Diagnosis of MIAC

MIAC was determined based on a positive PCR analysis for *Ureaplasma* species, *Mycoplasma hominis* and/or for *Chlamydia trachomatis* and/or by positivity for the 16S rRNA gene.

### Diagnosis of histological chorioamnionitis (HCA)

The degree of neutrophil infiltration was separately assessed in the free fetal membranes (amnion and chorion-decidua), placenta (amnion and chorionic plate), and umbilical cord according to the criteria of Salafia et al. [29]. A diagnosis of HCA was determined based on the presence of neutrophil infiltration in the chorion-decidua (grades 3–4), chorionic plate (grades 3–4), umbilical cord (grades 1–4), and/or amnion (grades 1–4) [29]. The diagnosis of funisitis was performed based on the presence of neutrophil infiltration in the umbilical cord (grades 1–4).

### Diagnosis of severe neonatal morbidity

Three investigators (IM, MS, and MK) reviewed the maternal and perinatal medical records. Data regarding the morbidity and mortality were reviewed for all newborns. For the current study, we defined “severe neonatal morbidity” as follows: the need for tracheal intubation; respiratory distress syndrome (defined by the presence of two or more of the following criteria: evidence of respiratory compromise, a persistent oxygen requirement for more than 24 hours, administration of exogenous surfactant, and radiographic evidence of hyaline membrane disease); intraventricular hemorrhage (diagnosed by cranial ultrasound examinations based on the criteria defined by Papile et al. [30]); necrotizing enterocolitis (defined as the radiologic finding of either intramural gas or free intra-abdominal gas); retinopathy of prematurity (identified using retinoscopy); early- and late-onset sepsis (defined as any systemic bacterial infection documented by a positive blood culture and/or the presence of symptoms of strong clinical suspicion of sepsis [the presence of symptoms and elevated C-reactive protein and/or an affected white blood cell count] during the first 72 hours of life and between 4 and 120 days of life, respectively); bronchopulmonary dysplasia (defined by the infant’s oxygen dependence at 36 weeks of age); pneumonia (diagnosed by abnormal findings on chest X-rays); and/or neonatal death prior to hospital discharge [31].

### Statistical analysis

The demographic characteristics were compared using the non-parametric Kruskal-Wallis test for continuous variables, and the data are presented as the medians (range). Categorical variables were compared using the Chi-square test or the Fisher’s exact test and are presented as percentages (%). For the analysis of the amniotic fluid IL-6 levels among the subgroups of PPROM, a non-parametric Kruskal-Wallis test was used. For the analysis of short-term neonatal morbidity among subgroups, the Fisher’s exact test was used. Spearman partial correlation was used to adjust the data for the gestational age at delivery. For the analyses among four subgroups, exact 2-tailed *p*-values were used due to the small sample size in the group with sterile IAI. All *p*-values were from two-sided tests, and all statistical analyses were performed using SPSS 19.0 (SPSS Inc., Chicago, IL, USA) and GraphPad Prism 5.03 for Mac OS X (GraphPad Software, La Jolla, CA, USA).

## Results

**Fig 1:**
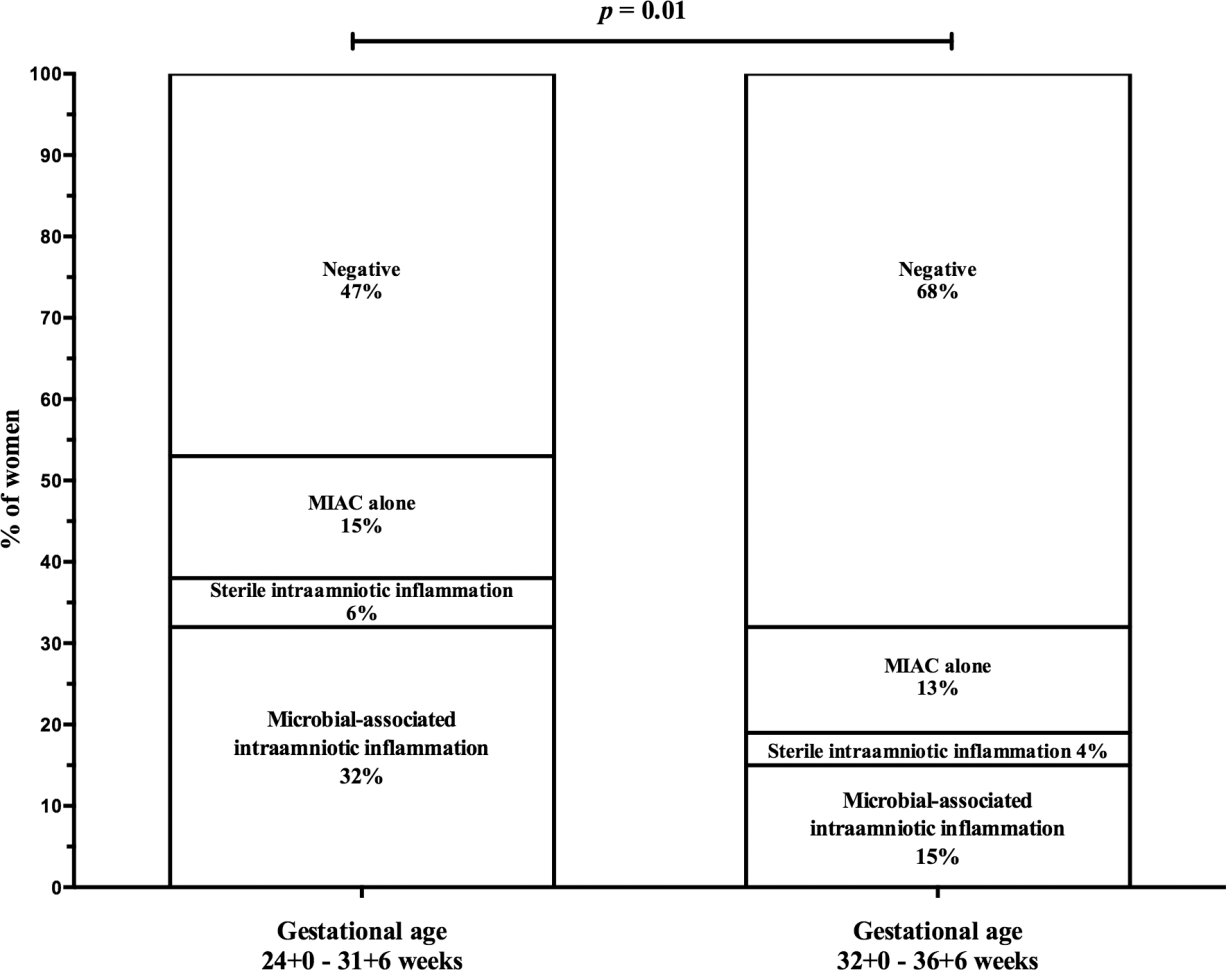
Prevalence of microbial-associated intraamniotic inflammation, sterile inflammation and microbial invasion of the amniotic cavity alone according to the gestational age at sampling.

In total, 166 women with PPROM at gestational ages 24+0 to 36+6 weeks were recruited. The demographic and clinical characteristics of the women are shown in Table 1. The overall rates of IAI and MIAC were 25% (41/166) and 34% (57/166), respectively. The presence of microbial-associated IAI (both IAI and MIAC) was observed in 21% (34/166) of women, 4% (7/166) had sterile IAI (IAI alone), 14% (23/166) had MIAC alone, and the remaining 61% (102/166) of the women were negative (without both MIAC and IAI). If the diagnosis of IAI was expanded to include both a positive IL-6 and neutrophil infiltration of the amnion (histological amnionitis), then the proportion of women in these four groups was 23%, 10%, 11% and 56%, respectively. Women with PPROM between gestational ages 24+0 and 31+6 weeks had a higher frequency of microbial-associated IAI [32% (17/53) vs. 15% (17/113)], almost the same frequencies of sterile IAI [6% (3/53) vs. 4% (4/113%)], almost the same frequency of MIAC alone [15% (8/53) vs. 13% (15/113)], and a lower frequency of negative women [49% (25/53) vs. 71% (77/113); *p* = 0.01; Fig 1] than women with PPROM between gestational ages 32+0 and 36+6 weeks (Table 2). Women with microbial-associated IAI were more likely to smoke and had higher C-reactive protein levels, white cell blood counts, and rates of HCA and funisitis compared to the remaining women (Table 1). Women with microbial-associated IAI also had newborns with lower birth weights. All of the microorganisms detected in the amniotic fluid are shown in Table 3. The most common bacteria were *Ureaplasma* spp., which were identified in 24% (40/166) of the women. Polymicrobial findings were observed in 11% (19/166) of women.

**Table 1:** Maternal and neonatal characteristics of preterm prelabor rupture of membrane pregnancies according to the presence or absence of microbial-associated intraamniotic inflammation, sterile intraamniotic inflammation and MIAC alone.

Characteristic	Microbial-associated IAI (n = 34)	Sterile IAI (n = 7)	MIAC alone (n = 23)	Negative (n = 102)	*p*-value^1^	*p*-value^2^
Maternal age	32 (17–42)	29 (21–36)	32 (18–39)	31 (21–42)	0.89	0.75
Prepregnancy body mass index [kg/m^2^, median (range)]	22.5 (16.5–38.0)	24.2 (19.3–25.7)	22.3 (17.6–33.5)	22.3 (15.8–39.0)	0.72	0.62
Smoking [number (%)]	16 (47%)	0 (0%)	5 (22%)	10 (10%)	**<0.0001**	**<0.0001**
Gestational age at admission [weeks, median (range)]	32+0 (24+2–36+6)	34+6 (25+1–36+6)	33+0 (25+3–36+6)	33+6 (24+5–36+5)	0.16	0.08
Gestational age at delivery [weeks, median (range)]	32+1 (24+5–36+6)	35+0 (25+1–36+6)	34+6 (26+6–36+6)	34+2 (25+2–36+5)	**0.04**	**0.02**
Latency from PPROM to amniocentesis [hours, median (range)]	8 (1–97)	7 (2–22)	6 (1–20)	5 (1–72)	0.11	0.06
Latency from amniocentesis to delivery [hours, median (range)]	30 (3–101)	40 (17–90)	37 (7–211)	28 (4–178)	0.66	0.77
Amniotic fluid IL-6 [pg/mL, median (range)]	4541 (804–10000)	837 (799–3073)	178 (50–327)	170 (50–729)	**<0.0001**	**<0.0001**
CRP levels at admission [mg/L, median (range)]	12.1 (0.6–106.0)	7.6 (1.0–16.6)	4.7 (0.1–23.0)	5.6 (0.5–44.4)	**0.002**	**0.001**
WBC count ad admission [x10^9^ L, median (range)]	13.8 (9.2–22.7)	12.1 (8.9–15.3)	11.9 (8.4–18.2)	11.5 (7.4–20.6)	**0.003**	**0.001**
Administration of antibiotics [number (%)]	34 (100%)	7 (100%)	23 (100%)	100 (98%)	1.00	0.57
Administration of corticosteroids [number (%)]	28 (82%)	6 (86%)	16 (70%)	74 (73%)	0.59	0.45
Vaginal delivery [number (%)]	23 (68%)	5 (71%)	17 (74%)	66 (65%)	0.88	0.69
Cesarean section [number (%)]	11 (32%)	2 (29%)	6 (26%)	36 (35%)	0.88	0.69
Birth weight [grams, median (range)]	1800 (550–3390)	2130 (990–3320)	2150 (780–3250)	2255 (700–3350)	**0.02**	**0.008**
Histological chorioamnionitis [number (%)]	32 (94%)	5 (71%)	15 (65%)	57 (56%)	**<0.0001**	**<0.0001**
Funisitis [number (%)]	22 (64%)	2 (29%)	9 (39%)	28 (27%)	**0.001**	**<0.0001**
Apgar score <7; 5 minutes [number (%)]	2 (6%)	1 (14%)	0 (0%)	3 (3%)	0.18	0.45
Apgar score <7; 10 minutes [number (%)]	1 (3%)	0 (0%)	0 (0%)	2 (2%)	1.00	0.72

Abbreviations: IAI: Intraamniotic inflammation, MIAC: microbial invasion of the amniotic cavity, IL-6: Interleukin 6, CRP: C-reactive protein, WBC: White blood cells. Statistically significant results are marked in bold. *p*-value: the comparison among four groups. Continuous variables were compared using the Kruskal-Wallis test (exact 2-tailed *p*-value). Categorical variables were compared using the Fisher’s exact test (exact 2-tailed *p*-value). *p*-value*: the comparison among women with microbial-associated intraamniotic inflammation, microbial invasion of the amniotic cavity alone, and negative women. Continuous variables were compared using the nonparametric Kruskal-Wallis test. Categorical variables were compared using a chi-square test.

**Table 2:** Prevalence of microbial-associated intraamniotic inflammation, sterile inflammation and microbial invasion of the amniotic cavity alone according to the gestational age at sampling.

Gestational age at sampling (weeks+days)	Microbial-associated IAI	Sterile IAI	MIAC alone	Negative
24+0–27+6 (n = 11)	5 (46%)	1 (9%)	2 (18%)	3 (27%)
28+0–31+6 (n = 36)	12 (29%)	2 (5%)	6 (14%)	22 (52%)
32+0–33+6 (n = 42)	5 (14%)	0 (0%)	4 (11%)	27 (75%)
34+0–36+6 (n = 77)	12 (16%)	4 (5%)	11 (14%)	50 (65%)

Abbreviations: IAI: Intraamniotic inflammation, MIAC: microbial invasion of the amniotic cavity.

**Table 3:** Microorganism identified in the amniotic fluid of women with preterm prelabor rupture of membranes.

The presence of microbial-associated intraamniotic inflammation (n = 34)	The presence of microbial invasion of the amniotic cavity alone (n = 23)
*Ureaplasma* species	*Ureaplasma* species
*Ureaplasma* species	*Ureaplasma* species
*Ureaplasma* species	*Ureaplasma* species
*Ureaplasma* species	*Ureaplasma* species
*Ureaplasma* species	*Ureaplasma* species
*Ureaplasma* species	*Ureaplasma* species
*Ureaplasma* species	*Ureaplasma* species
*Ureaplasma* species	*Ureaplasma* species
*Ureaplasma* species	*Ureaplasma* species
*Ureaplasma* species	*Ureaplasma* species
*Ureaplasma* species	*Ureaplasma* species
*Ureaplasma* species	*Mycoplasma hominis*
*Streptococcus agalactiae*	*Mycoplasma hominis*
*Streptococcus anginosus*	*Mycoplasma hominis*
*Haemophilus influenzae*	*Mycoplasma hominis*
*Staphylococcus hominis*	*Bifidobacterium* species
*Streptococcus species*	*Staphylococcus haemolyticus*
*Chlamydia trachomatis*	*Enterococcus faecium*
*Ureaplasma* species + *Mycoplasma hominis*	*Chlamydia trachomatis*
*Ureaplasma* species + *Mycoplasma hominis*	*Gardnerella vaginalis*
*Ureaplasma* species + *Mycoplasma hominis*	*Ureaplasma* species + *Chlamydia trachomatis*
*Ureaplasma* species + *Mycoplasma hominis*	*Ureaplasma* species + *Haemophilus influenzae*
*Ureaplasma* species + *Mycoplasma hominis*	*Ureaplasma* species + *Chlamydia trachomatis*
*Ureaplasma* species + *Veillonella* species	
*Ureaplasma* species + *Lactobacillus* species	
*Ureaplasma* species + *Sneathia sanguinegens*	
*Ureaplasma* species + *Chlamydia trachomatis*	
*Ureaplasma* species + *Sneathia sanguinegens*	
*Ureaplasma* species + *Chlamydia trachomatis*	
*Ureaplasma* species + *Streptococcus anginosus*	
*Ureaplasma* species + *Haemophilus influenzae*	
*Ureaplasma* species + *Sneathia sanguinegens*	
*Chlamydia trachomatis + Leptotrichia amnionii*	
*Peptococcus species + Propionibacterium species + Bacteroides species*	

**Fig 2:**
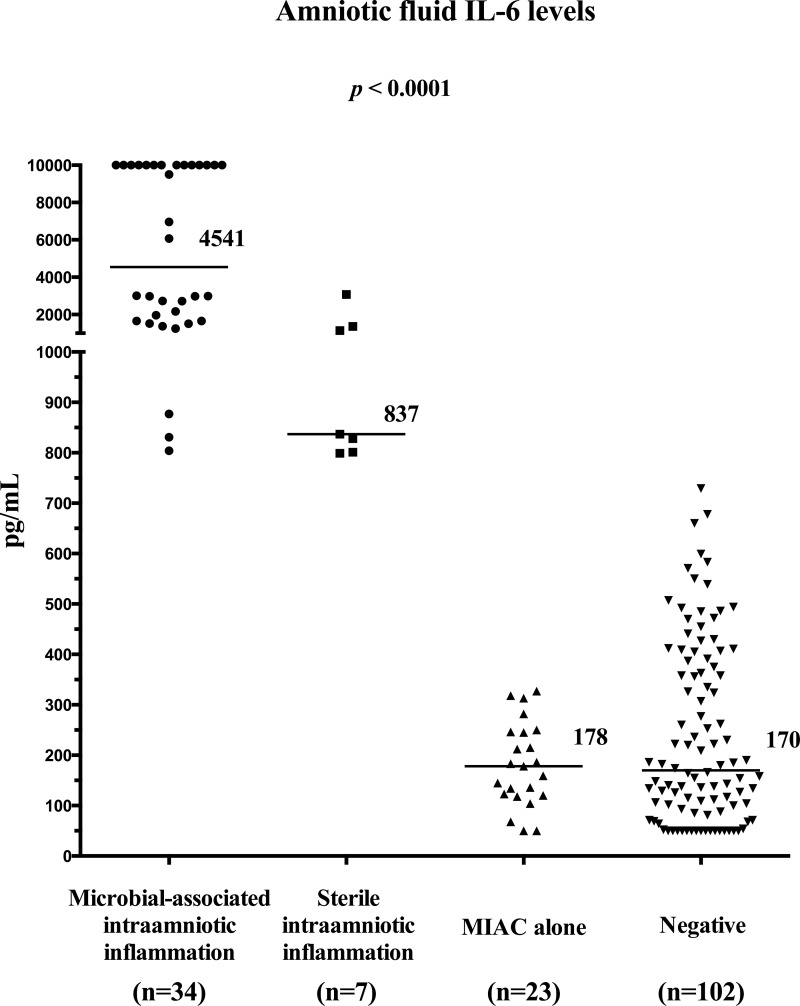
Amniotic fluid interleukin-6 concentrations in preterm prelabor rupture of membrane pregnancies that are complicated with the presence or absence of microbial-associated intraamniotic inflammation, sterile intraamniotic inflammation and microbial invasion of the amniotic cavity alone.

Women with microbial-associated IAI had higher median amniotic fluid IL-6 levels than the remaining women (with microbial-associated IAI: median 4541 pg/mL [range: 804–10000] vs. with sterile IAI: median 837 pg/mL [range 799–3073] vs. with MIAC alone: median 178 pg/mL [range 50–327] vs. negative: median 170 [range 50–729] pg/mL; *p*<0.0001; Fig 2).

**Fig 3:**
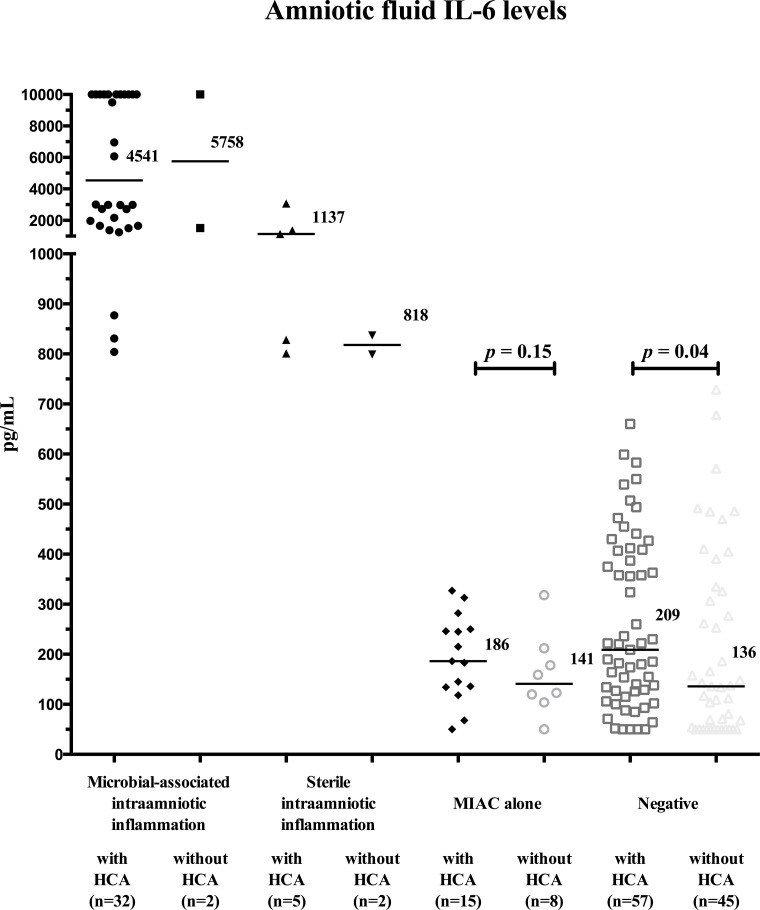
Amniotic fluid interleukin-6 concentrations in preterm prelabor rupture of membrane pregnancies that are complicated with the presence or absence of microbial-associated intraamniotic inflammation, sterile intraamniotic inflammation and microbial invasion of the amniotic cavity alone with respect to the presence and absence of histological chorioamnionitis.

**Fig 4:**
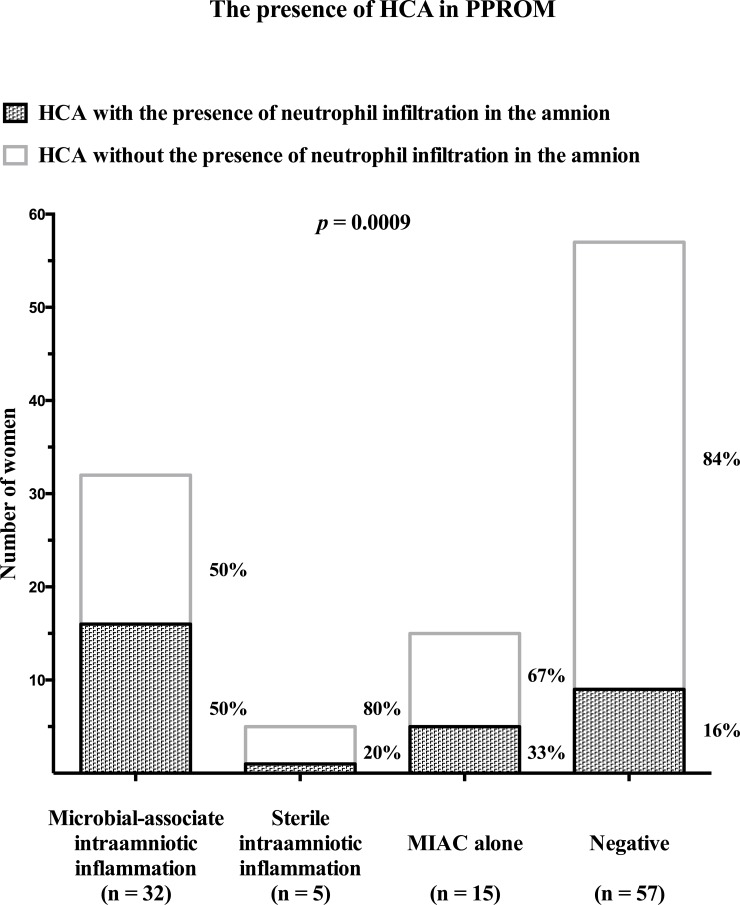
Prevalence of histological chorioamnionitis with and without the presence of neutrophil infiltration in the amnion in women with microbial-associated intraamniotic inflammation, sterile intraamniotic inflammation and microbial invasion of the amniotic cavity alone.

The presence of HCA was observed in 94% (32/34) of women with microbial-associated IAI, in 71% (5/7) of women with sterile IAI, in 65% (15/23) of women with MIAC alone, and in 56% (57/102) of negative women. Funisitis was found in 68% (23/34) of women with microbial-associated IAI, in 29% (2/7) of women with sterile IAI, in 35% (8/23) of women with MIAC alone, and in 27% (28/102) of negative women. Women with MIAC alone did not have higher amniotic fluid IL-6 levels when HCA present (with HCA: median 186 pg/mL vs. without HCA: median 141 pg/mL; *p* = 0.15; Fig 3). Negative women had higher amniotic fluid IL-6 levels when HCA was present (with HCA: median 209 pg/mL vs. without HCA: median 136 pg/mL; *p* = 0.04; Fig 3). Women with microbial-associated IAI had a higher rate of HCA with neutrophil infiltration of the amnion than the remaining women [with microbial-associated IAI: 50% (16/32) vs. with sterile IAI: 20% (1/5) vs. with MIAC alone: 33% (5/15) vs. negative 16% (9/57); *p* = 0.0009; Fig 4). The grades of neutrophil infiltration in the chorion-decidua, the chorionic plate, the umbilical cord and the amnion in all subgroup of women are shown in S1 Table.

**Fig 5:**
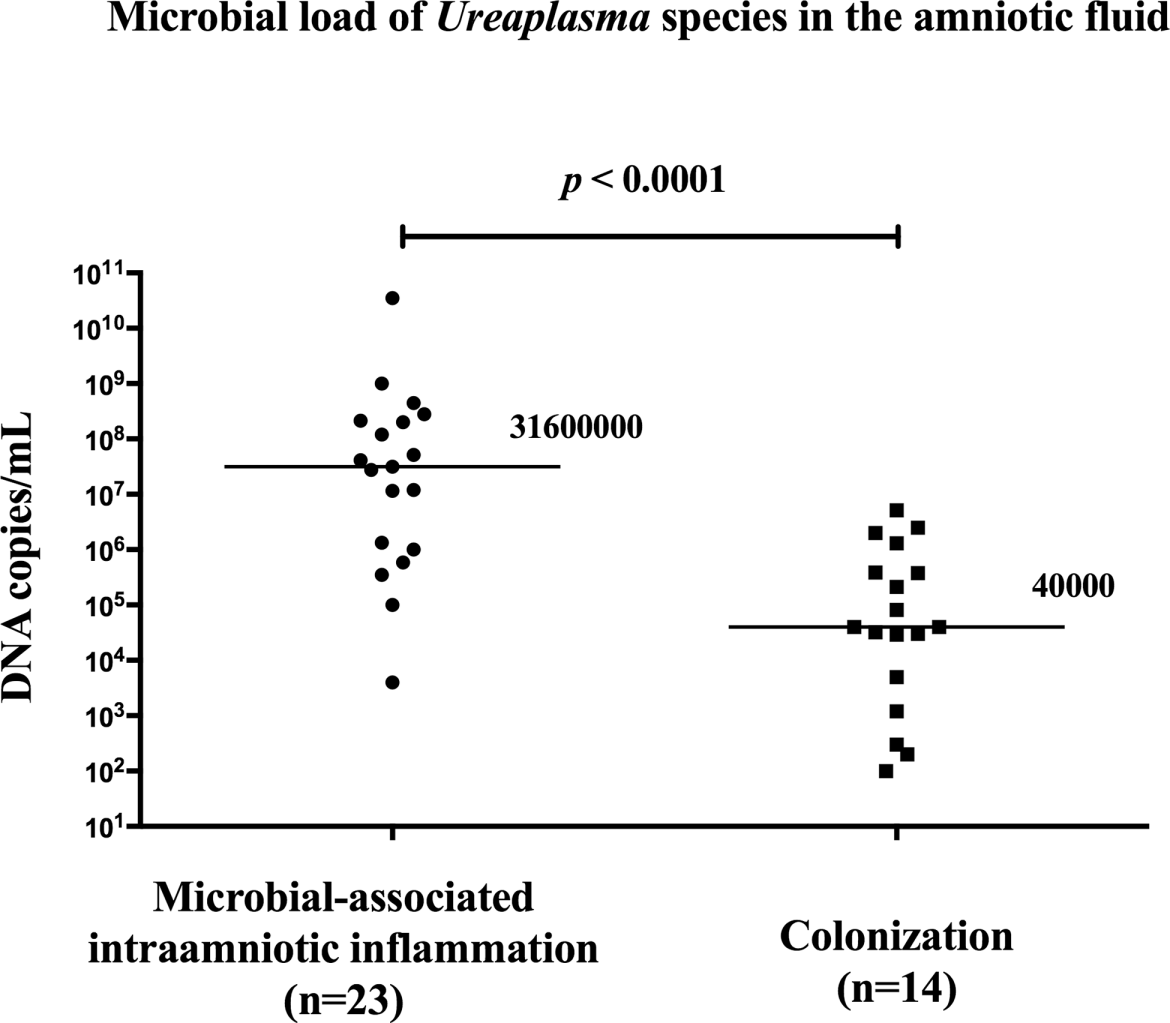
Amniotic fluid microbial loads of *Ureaplasma* species in preterm prelabor rupture membrane pregnancies that are complicated by the microbial-associated intraamniotic inflammation and microbial invasion of the amniotic cavity alone.

**Fig 6:**
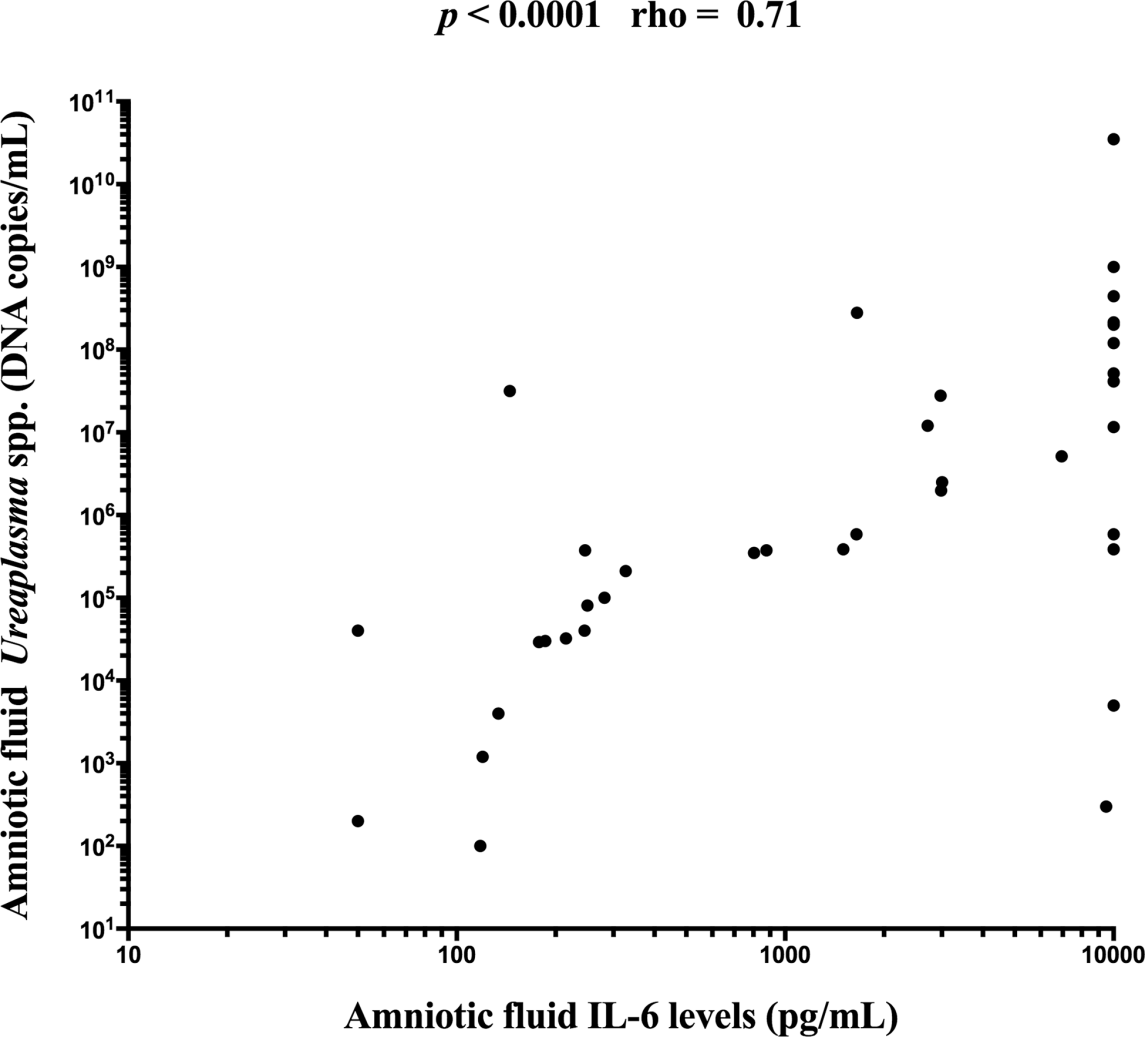
A correlation between microbial load of *Ureaplasma* species and interleukin-6 in the amniotic fluid from pregnancies complicated by preterm prelabor rupture of membranes.

Women with the presence of microbial-associated IAI had a higher load of *Ureaplasma* species in the amniotic fluid than women with MIAC alone (with microbial-associated IAI: median 3.2x10^7^ copies DNA/mL [range 4.0x10^3^-.3.5x10^10^] vs. MIAC alone: median 4.0x10^4^ copies DNA/mL [range 1.0x10^2^-5.1x10^6^]; *p* < 0.0001; Fig 5). There was a strong correlation (rho 0.71; *p* < 0.0001) between microbial load of *Ureaplasma* species and IL-6 in the amniotic fluid (Fig 6).

When selected indicators of short-term neonatal morbidity were examined, differences in the rates of the need for tracheal intubation and bronchopulmonary dysplasia were found in the crude analysis. However, there were no differences in the rates of the selected aspects of short-term neonatal morbidity after adjusting for gestational age at delivery (Table 4).

**Table 4:** Neonatal morbidity among preterm prelabor rupture of membrane pregnancies according to the presence or absence of microbial-associated intraamniotic inflammation, sterile intraamniotic inflammation and microbial invasion of the amniotic cavity alone.

Characteristic	The presence of microbial-associate IAI (n = 34)	The presence of sterile IAI (n = 7)	The presence of MIAC alone (n = 23)	Negative (n = 102)	*p*-value^1^	*p*-value^2^	*p*-value^3^	*p*-value^4^
Tracheal intubation [number (%)]	3 (9%)	1 (14%)	0 (0%)	4 (4%)	0.19	0.72	0.26	0.77
Respiratory distress syndrome [number (%)]	13 (38%)	2 (29%)	9 (39%)	25 (25%)	0.30	0.75	0.18	0.69
Bronchopulmonary dysplasia [number (%)]	4 (12%)	1 (14%)	2 (9%)	2 (2%)	**0.03**	0.26	**0.05**	0.29
Intraventricular hemorrhage [number (%)]	4 (12%)	1 (14%)	4 (17%)	12 (12%)	0.81	0.52	0.75	0.57
Necrotizing enterocolitis [number (%)]	2 (6%)	0 (0%)	0 (0%)	1 (1%)	0.27	0.33	0.19	0.32
Early-onset sepsis [number (%)]	2 (6%)	0 (0%)	2 (9%)	2 (2%)	0.26	0.65	0.27	0.60
Pneumonia [number (%)]	2 (6%)	0 (0%)	0 (0%)	1 (1%)	0.27	0.28	0.15	0.27
Neonatal death before hospital discharge [number (%)]	0 (0%)	0 (0%)	0 (0%)	3 (3%)	0.77	0.16	0.43	0.17
Severe neonatal morbidity [number (%)]	16 (47%)	2 (29%)	9 (4%)	34 (33%)	0.51	0.86	0.35	0.97

Abbreviations: IAI: Intraamniotic inflammation, MIAC: microbial invasion of the amniotic cavity. Severe neonatal morbidity was defined as a need for intubation and/or respiratory distress syndrome and/or pneumonia and/or bronchopulmonary dysplasia and/or intraventricular hemorrhage and/or necrotizing enterocolitis and/or early-onset sepsis and/or late-onset sepsis and/or neonatal death before hospital discharge. Retinopathy of prematurity (n = 1) and late onset sepsis (n = 2) was not considered in the analysis because of low occurrence in the cohort. Categorical variables were compared using the Fisher’s exact test or the chi-square test. Spearman partial correlation was used to adjust the data for the gestational age at delivery. Statistically significant results are marked in bold. *p*-value^1^: the comparison among four groups with the Fisher’s exact tests (exact 2-tailed *p*-value). *p*-value^2:^ the comparison among four groups with the adjustment for gestational age at delivery. *p*-value^3^: the comparison among women with microbial-associated intraamniotic inflammation, microbial invasion of the amniotic cavity alone, and negative women with the chi-square test. *p*-value^4^: the comparison among women with microbial-associated intraamniotic inflammation, microbial invasion of the amniotic cavity alone, and negative women with the adjustment for gestational age at delivery.

## Discussion

Pregnancies with PPROM are often complicated by IAI and MIAC. The management of PPROM mainly depends on the gestational age, severity of the clinical presentation and/or knowledge about the complications. Therefore, a prompt diagnosis of these complications seems to be important for the optimal management of PPROM, appropriate parental counseling and providing adequate neonatal care. Ideally, this information should be available within a short time from the admission of women with PPROM. However, the clinical dilemma for initiating appropriate management is attributed to the lack of proper biochemical or clinical indicators. In this study, we examined subgroups of women with PPROM based on the presence or absence of IAI and MIAC. We used an AF IL-6 value of >750 pg/ml as the definition of IAI based on its prognostic utility in women presenting with PPROM [24].

The five principal findings of this study are as follows: i) microbial-associated IAI was a complication in approximately one-fifth of women with PPROM; ii) sterile IAI is rare in PPROM; iii) microbial-associated IAI was associated with a higher rate of neutrophil infiltration of the amnion iv) microbial-associated IAI was related to a higher microbial load of *Ureaplasma* species in the amniotic fluid compared to MIAC alone; and v) there were no differences in the short-term neonatal morbidity with respect to the presence of microbial-associated IAI, sterile IAI, and MIAC alone after adjusting for gestational age at delivery in women with PPROM.

In this study, we found that approximately 21% of PPROM was complicated by IAI. The majority of these cases (83%; 34/41) were complicated by microbial-associated IAI. Sterile IAI was responsible for a small proportion of the pregnancies complicated by IAI (17%; 7/41). This finding is different from the data reported by Romero et al. in which approximately one-third of women with PPROM had sterile inflammation. In this Romero et al. study where amniotic fluid IL-6 was evaluated by ELISA cut-off level for IAI was 2.6 ng/mL. However, recent studies by Chaemsaithong et al. have shown that 0.745 pg/mL (cut-off used in this study) is equivalent of this cut-off when amniotic fluid IL-6 is measured by a point of care test (used in this study) [24]. It means that the definition for IAI was the same in ours and Romero et al. studies. Different factors may explain the discrepancy between the studies. First, the difference in the gestational ages of PPROM could contribute to the discrepancy. In our study, only women with PPROM and gestational ages between 24+0 and 36+6 weeks were included, but in Romero’s et al. study, women with gestational ages between 20 and 35 weeks were included. Forty-two percent of women in our study and 90% of women in Romero’s study were at gestational age < 33 weeks upon sampling of PPROM. Secondly, the participants’ ethnicities and geographic backgrounds differed between the studies, and amniotic fluid IL-6 has been documented to demonstrate ethnic variations. We do not want to overestimate the rate of sterile IAI in Caucasian woman with pregnancies complicated by PPROM between gestational ages 24 and 37 weeks. Nevertheless, this finding seems to be clinically relevant because only the evaluation of amniotic fluid IL-6 levels, without an evaluation of the MIAC, can identify women at high risk of the presence of microbial-associated IAI. This is clinically relevant because the diagnosis of IAI is relatively easy to perform with evaluation of the amniotic fluid IL-6 using broadly available point of care tests. Moreover, the diagnosis of MIAC requires more time and the availability of a laboratory that can perform non-culture-based bacteria detection tests or at least has specific cultivation techniques for genital mycoplasmas. While, these findings are clinically relevant and have important clinical implications, we are aware that these findings must be validated on a new independent cohort of women.

The neutrophil infiltration of the amnion is considered as the most advanced stage of HCA related to the highest intraamniotic and fetal inflammatory response [32]. In this study we found that women with microbial-associated IAI had a higher rate of neutrophil infiltration of the amnion than the remaining women with PPROM. It means that this subgroup of PPROM is more often complicated by the most advanced stage of HCA. In addition, it has been shown that the presence the neutrophil infiltration of the amnion is a better indicator of the development of early-onset sepsis than the presence of funisitis [32]. This finding is in line with our observations because all pregnancies complicated by the development of early onset sepsis had the neutrophil infiltration of the amnion.

Genital mycoplasmas (*Ureaplasma* species and *Mycoplasma hominis)* are the most common microorganisms isolated from amniotic fluid obtained from pregnancies complicated by PPROM. In the subgroup of women with microbial-associated IAI, *Ureaplasma* species either alone or with other microorganisms was found in 10 women and in 16 women, respectively. In remaining 5 women, *Streptococcus anginosus*, *Streptococcus* species, *Haemophilus influenzae*. *Staphylococcus hominis*, and *Chlamydia trachomatis* were found. In the subgroup of women with MIAC alone, only *Ureaplasma* species and only *Mycoplasma hominis* was found in 13 and 4 women, respectively. Three women had the presence of *Ureaplasma* species with other bacteria, and the remaining 6 women had *Gardnerella vaginalis*, *Bifidobacterium* species, *Streptococcus agalactiae*, *Staphylococcus haemolyticus*, *Enterococcus faecium* and *Chlamydia trachomatis* in the amniotic fluid. Our previous studies have shown that the intensity of intraamniotic inflammation correlates with the microbial load of genital mycoplasmas [12,13]. In this study, there was a higher microbial load of *Ureaplasma* species in the amniotic fluid of women with microbial-associated IAI than in women with MIAC alone This finding confirms the hypothesis that the intraamniotic inflammatory response of the amniotic cavity determined in the presence of MIAC is likely related to the microbial load and type of bacteria. In this study we confirmed our previous observation that intraamniotic inflammatory response to *Ureaplasma* species is a dose-dependent [12,33].

From a clinical point of view, it is also important to understand the association between the microbial-associated IAI, sterile IAI, MIAC alone and short-term neonatal morbidity. We did not find any evidence suggesting that the presence of these conditions is detrimental to the short-term neonatal outcomes of newborns from PPROM after adjusting for the gestational age at delivery. Therefore, the gestational age at delivery but not the presence of microbial-associated IAI, sterile IAI or MIAC alone is an important factor for neonatal morbidity. This is in line with previous reports showing that the gestational age both at PPROM and delivery is associated with adverse neonatal outcomes [34,35]. Moreover, a recent paper by Combs et al. reported similar findings in a cohort of women with spontaneous preterm delivery with intact membranes [15]. Our findings confirm that expectant management is justified in pregnancies complicated by PPROM. However, the association between the presence of microbial-associated IAI, sterile IAI, MIAC alone and long-term neonatal outcomes (e.g., cerebral palsy and neurodevelopmental outcomes) should be determined.

An important strength of this study is that MIAC was identified based on non-cultivation techniques, including non-specific PCR (16S rRNA) and specific PCR (*Ureaplasma* species, *Mycoplasma hominis*, and *Chlamydia trachomatis*). The implementation of specific PCR gave us the opportunity to quantify the microbial load of *Ureaplasma* species in amniotic fluid. The second strength is that only women with one well-defined phenotype of preterm delivery (PPROM, defined as a rupture of the fetal membranes with a leakage of the amniotic fluid at least two hours before the onset of regular uterine activity) were included in this study. The main limitation of this study is the relatively small sample size. We also used only non-culture DNA-based methods in this study that were able to detect bacterial DNA from the majority of cases with MIAC, although it should be noted that the DNA analysis method cannot distinguish between live and dead bacteria. A further limitation of the study is the lack of information on *Ureaplasma* species or serovars, which would be helpful for the establishment of a more complex understanding of the association between these microorganisms, PPROM, IAI and short-term neonatal outcomes.

In conclusion, microbial-associated IAI is more common in PPROM, and sterile IAI is rare in Caucasian women with PPROM between gestational ages 24–37 weeks. Evaluation of the amniotic fluid IL-6 without identifying the bacteria in the amniotic fluid can be used to identify PPROM complicated by microbial-associated IAI. The presence of microbial-related IAI or sterile IAI does not affect short-term neonatal outcomes in PPROM.

## Supporting Information

S1 Table Amniotic fluid IL-6 levels, the presence of histological chorioamnionitis, funisitis, and the presence of neutrophil infiltration (grades 1–4) in the chorion-decidua, chorionic plate, umbilical cord, and amnion in preterm prelabor rupture of membrane pregnancies that are complicated with the presence or absence of microbial-associated intraamniotic inflammation, sterile intraamniotic inflammation and microbial invasion of the amniotic cavity alone. (XLSX)
